# Serum amyloid-p (SAP), a potential biomarker for Down syndrome fetuses prevention in maternal plasma

**DOI:** 10.1186/1878-5085-5-S1-A98

**Published:** 2014-02-11

**Authors:** Athanasios Anagnostopoulos, George Th Tsangaris

**Affiliations:** 1 Proteomics Research Unit, Center of Basic Research II, Biomedical research Foundation of the Academy of Athens, Athens, Greece

## 

Down syndrome is the most common chromosomal abnormality in pregnancy. Non-invasive prenatal diagnosis (NIPD) of DS screening in the first trimester of pregnancy involves the nuchal transclucency hyperechographic scanning combined to the analysis of β-subunit of human chorionic gonadotropin (β-hCG) and pregnancy associated plasma protein-A (PAPP-A). Application of proteomics for the identification of potential biomarkers for the prenatal screening of DS revealed a group of proteins (AMBP, SAP, CERU, CLUS, APOE, AFAM, TTHY) differentially expressed during the 15^th^ week of pregnancy in the maternal plasma of women carrying DS fetuses compared to normal pregnancies [1,2]. These proteins could act as potential markers for the prevention of DS by NIPD. From this protein group we selected SAP because the specific protein seems to be the most promising for the NIPD of DS, since several studies have indicated SAP involvement to Alzheimer’s disease, a phenotype close to DS. In the present study, through western blot analysis we evaluated the capability of SAP to act as a reliable biomarker for the NIPD of DS fetuses at the 10^th^ -12^th^ and 15^th^ -16^th^ week of pregnancy [3-5]. We analyzed 25 serum samples from women carrying DS fetuses and 75 samples of women with normal fetuses at the 10^th^-12^th^ week of pregnancy. Furthermore SAP levels were evaluated in 12 plasma samples from women with DS fetuses and 15 samples of normal pregnancies at the 15^th^-16^th^ week of pregnancy. At the10^th^ - 12^th^ week of pregnancy we found that SAP levels were elevated in 24 out of the 25 DS pregnancies compared to normal controls. At the 15^th^ -16^th^ week of pregnancy SAP was increased in all the DS pregnancies compared to normal controls. Quantitative densitometric analysis indicated that at the 10^th^-12^th^ week of pregnancy SAP was increased by a mean of 20% in DS pregnancies compared to controls while at the 15^th^ - 16^th^ week of pregnancy SAP was increased by a mean of 40%. These results indicate that SAP could be a reliable marker for the prevention of DS fetuses in maternal plasma early at pregnancy.
